# Parameter adaptive sliding mode trajectory tracking strategy with initial value identification for the swing in a hydraulic construction robot

**DOI:** 10.1038/s41598-023-30952-x

**Published:** 2023-04-11

**Authors:** Jing-Wei Hou, Tao Ni, Zhu-Xin Zhang

**Affiliations:** 1 School of Mechanical and Aerospace Engineering, Jilin University, Changchun, 130022 China; 2 School of Mechanical Engineering, YanShan University, Qinghuangdao, 066004 China

**Keywords:** Electrical and electronic engineering, Mechanical engineering

## Abstract

A novel trajectory tracking strategy is developed for a double actuated swing in a hydraulic construction robot. Specifically, a nonlinear hydraulic dynamics model of a double actuated swing is established, and a parameter adaptive sliding mode control strategy is designed to enhance the trajectory tracking performance. When an object is grabbed and unloaded, the moment of inertia of a swing considerably changes, and the performance of the estimation algorithm is generally inadequate. Thus, it is necessary to establish an algorithm to identify the initial value of the moment of inertia of the object. To this end, this paper proposes a novel initial value identification algorithm based on a two-DOF robot gravity force identification method combined with stereo vision information. The performance of the identification algorithm is enhanced. Simulations and experiments are performed to verify the effect of the novel control scheme.

## Introduction

Due to the risks associated with the presence of operating errors and dynamic changes in the environment, the development of fully automated systems for robotic construction machines or construction robots is limited, although automatic control can help achieve a high precision and efficiency for many kinds of machines^1,2^. To promote the operation of robotic construction machines, it is necessary to enhance the environmental perception and implement intelligent assistance with automatic control for novice operators^3–5^. By optimizing the proportional integral derivative (PID) controller, Feng et al.^6^ proposed an improved ant colony optimization algorithm (IACO) to increase the tracking accuracy of hydraulic systems. Moreover^7^, a robust controller was designed using the µ-synthesis method to ensure the stability and performance of a hydraulic excavator.

Zhao et al. developed a construction robot system with physical human–robot interaction (PHRI), which could perform tasks in earthquake disaster sites^8,9^. In such systems, as the end effector operation may be dangerous to the operator, a novel force strategy associated with a master–slave control schematic is usually adopted.

Most engineering robot systems are actuated through hydraulic servo systems that suffer from disturbances and uncertainties. To attain high-performance trajectory tracking control in uncertain nonlinear systems, the robust adaptive control method^10^ has been widely used because of its flexibility and robustness. For example^11^, a fuzzy logic controller was designed to track the trajectory of an industrial robot with 2 degrees of freedom. Three particle swarm optimization algorithms with different cost functions were used to optimize the controller parameters. The fuzzy logic in the algorithm decreased the complexity of mathematical modelling, which is commonly a complex and time-consuming process. However, owing to the lack of comprehensive analyses, the algorithm lacked generality in multiple situations, even though satisfactory results were obtained in the specific experiments. Among such strategies, adaptive sliding mode control is a valuable control method^12–14^.

Adaptive sliding mode control is a new control strategy for nonlinear systems. This method combines the advantages of adaptive control and sliding mode control by introducing adaptive estimation into the sliding mode controller. According to the information uncertainty determined using the adaptive controller, the sliding mode controller can be adjusted to decrease the system uncertainty and conservativeness of the sliding mode control. In this manner, the system can maintain the robustness of sliding mode control to external disturbances and unmodelled dynamics, and the adaptive control strategy can help overcome the limitations of sliding mode control^15–18^. To suppress the motion disturbance of the actuator, a nonlinear robust dual-loop control scheme was proposed^19^. In addition to considering the motion disturbance of the actuator, the nonlinear characteristics and friction problems of the EHLS were considered. Furthermore, a continuous control set model predictive speed control (CCS-MPSC) based on the fast terminal cost index (FTCI) was proposed^20^ to enhance the system tracking performance.

Although sliding mode control is highly robust, it is based on the precise mathematical model of the object^21–24^. The “rough” mathematical model of the system must be predicted before the system sliding mode surface parameters and controller parameters can be determined. For nonlinear systems, the dynamics of the nonlinear function of the system must be identified before designing the sliding mode controller. The method of robot parameter identification to obtain accurate model parameters is of significance in the design of robot controllers including sliding mode controllers. To address the uncertain load disturbances of a hydraulic Stewart manipulator, sliding mode control based on the discontinuous projection adaptive law was developed to enhance the tracking performance^25–27^. Cao et al. used neural networks to adjust the switching gain online and realize system identification and parameter prediction. Moreover, the authors designed a neural network sliding mode controller for hybrid electric vehicles^28^. Using the Szász–Mirakyan operator as the basic function and by adjusting the polynomial coefficients through the adaptive law determined through stability analyses, a robust adaptive controller was designed to enhance the impedance control of a robot manipulator^29^. Other common methods to enhance the estimation performance include the use of neuro-fuzzy model auxiliary filters and time-varying parameters^30–34^.

The aim of this article is about a novel method to identificate the moment of inertia for a robot with stereo vision and robot dynamic and its application for the adaptive sliding mode control strategy of a construction robot. A satisfactory trajectory tracking controller must ensure that a robot can perform precise trajectory tracking. To this end, the dynamic characteristics of the robot must be considered^35–38^. To design the controller for a swing when a robot carries an object and increase the trajectory tracking accuracy, it is necessary to design a robust adaptive controller to address the uncertainty of the parameters. For a hydraulic cylinder system, the load equivalent mass is the most important element in controller design. However, for a swing, because the load mass is large and changes in real time according to the working conditions, the equivalent mass cannot be accurately obtained. Nevertheless, it can be assumed that the load does not change during the carrying process. Based on the inverse dynamic analysis of the robot arm and boom cylinders, the initial value of the equivalent mass can be obtained through system identification^39^. Subsequently, relatively accurate initial values of the controller parameters can be obtained, and the convergence and trajectory tracking accuracy of the controller can be enhanced.

According to the former issues, the following work was complemented: A novel nonlinear hydraulic dynamics model is established for the double actuated hydraulic cylinder system of the swing in a hydraulic robot and a sliding mode control strategy with parameter adaptive estimation is designed to improve the trajectory tracking performance. A method for estimating the initial value of the moment of inertia based on stereo vision and robot dynamics is proposed in this manuscript. The estimated initial value of the moment of inertia is used in the robust adaptive control strategy to improve the trajectory tracking performance which contributes a novel strategy SMI (sliding mode control with initial value). The main contribution of the paper includes: A method obtaining the inertial moment parameters of the robot by using stereo vision and robot dynamics is proposed. Through the discussion of the initial value of the nonlinear adaptive algorithm of swing of a new type of engineering robot, the effect of this method in improving the trajectory tracking accuracy during the robot grasping and releasing process is proved. Some specific problems in the use of this method are discussed, such as the effect of the combination of the moment of inertia parameter acquisition method and PD controller, the parameter convergence characteristics of the parameter adaptive method, and the advantages of this method compared with the results obtained by the robot dynamics method only and stereo vision only.

The paper is organized as follows: The background and related research are described in "Introduction" section. "System and Problem Description" section presents the system overview of the teleoperation system and problem description. "Sliding adaptive robust controller for the swing" section describes the sliding mode adaptive robust controller for the swing. The method to identify the system initial value is described in "Identification of the moment of inertia" section. The simulation experiment is described in "Simulation experiment" section. "Experiment" section describes the experiment performed to compare the performance of three controllers carrying objects between two points.

## System and problem description

### System description

**Figure 1:**
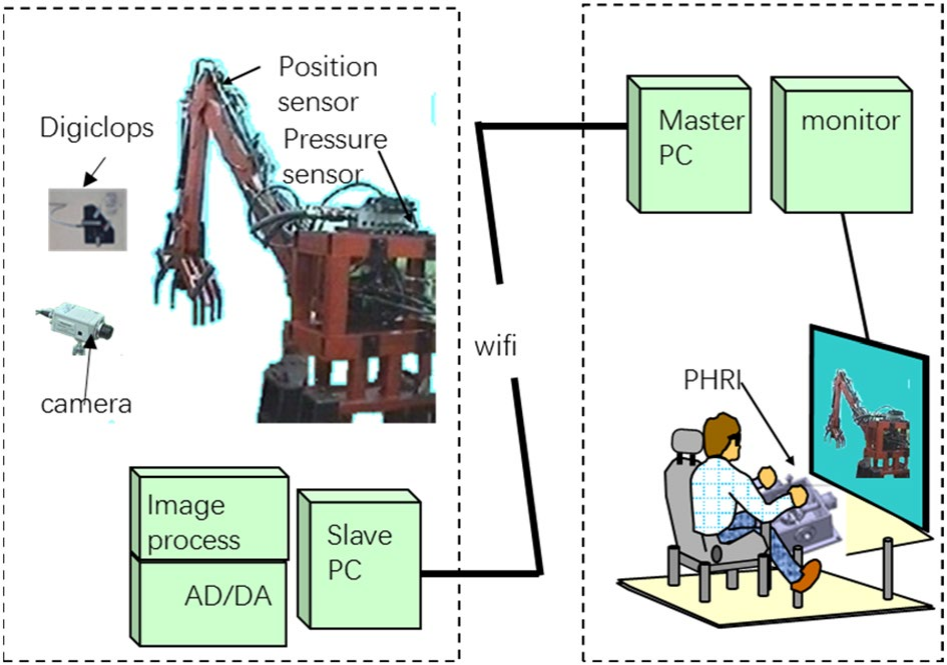
Teleoperation system.

The proposed teleoperation system consists of two parts: a pair of hydraulic PHRIs as the master and an engineering robot reconstructed from a hydraulic excavator as the slave, as shown in Fig. 1. Moreover, the system includes a control PC for each part.

The four asymmetric hydraulic cylinders are driven by servo valves. The working pressure can be measured using pressure sensors installed in the rod and rod-less cavities of the hydraulic cylinder. The displacement of the working device can be determined using a displacement sensor installed outside the hydraulic cylinder.

The system uses cameras for monitoring. However, considering the limitations of the camera viewing angle and resolution, a 3D camera is used to construct a virtual scene and the work object. The general shape of the object can be determined through the 3D camera, although the volume and shape cannot be accurately identified due to the low camera resolution.

**Figure 2:**
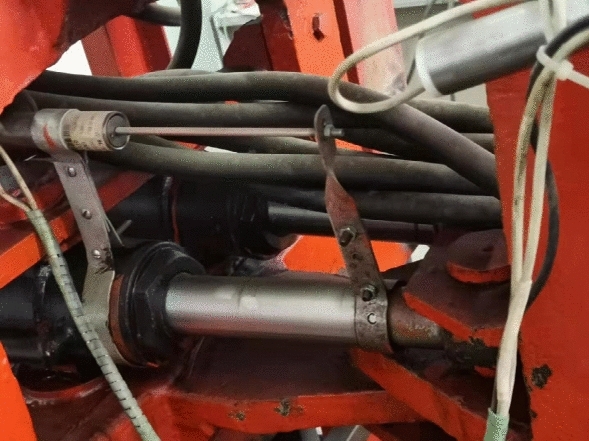
Swing DOF in the construction robot.

Figure 2 shows the actuation system of the swing. The double-cylinder actuated swing occupies a small space and does not need to drive the heavy chassis. The production cost of the structure increases because of the use of the double cylinders. However, because multi-point transmission and effective force transmission can be realized by the hydraulic system, this design exhibits a low energy consumption and compact structure.

### Problem description

The moment of inertia parameter of the swing varies considerably when the work object is carried and unloaded. However, the parameter has a constant value during one procedure. This article designs a sliding adaptive robust trajectory tracking controller for the swing of a hydraulic construction robot. However, the strategy suffers from a low convergence speed when the moment of inertia parameter varies. Therefore, a moment of inertia identification algorithm is used.

The main problem can be divided into the following subproblems.

1. According to the dynamic characteristics of the robot, the gravity compensation algorithm is used to determine the weight of the object according to the information provided by the arm and boom force sensors. The shape and size information of the working object is obtained through computer vision. These data points are combined with the weight information to determine the inertia of the object relative to the swing.
2. To effectively suppress the uncertainties and disturbances, a sliding mode adaptive controller is designed for the hydraulic servo system of the swing. By combining the control strategy and initial value obtained by determining the moment of inertia, the parameter convergence and trajectory tracking performance can be enhanced.

## Sliding adaptive robust controller for the swing

### Mathematical model of the swing

The dynamic model for the hydraulic system shown in Fig. 2 is

1 $$F_{1} l_{1} \sin \theta_{1} + F_{2} l_{2} \sin \theta_{2} = m{\ddot{l}}_{1} + I{\ddot{\theta }} + m{\ddot{l}}_{2} + C{\dot{\theta }}$$

where m is the mass of the cylinder rod, I is the equivalent mass of the robotic arm and the cylinders, F_1_ and F_2_ denote the load forces of the cylinders, and C is the damping coefficient for swing.

In this case,

2 $$\begin{aligned} & \left( {P_{1} A_{1} - P_{2} A_{2} } \right)l_{1} \sin \theta_{1} + \left( {P_{1} A_{2} - P_{2} A_{1} } \right)l_{2} \sin \theta_{2} \\ & \quad = m\ddot{l}_{1} + I\ddot{\theta } + m\ddot{l}_{2} + {\text{C}}\dot{\theta } \\ \end{aligned}$$

where A_1_ and A_2_ are the areas of the two sides of the piston, and P_1_ and P_2_ denote the pressures in the two chambers of the cylinder.

**Figure 3:**
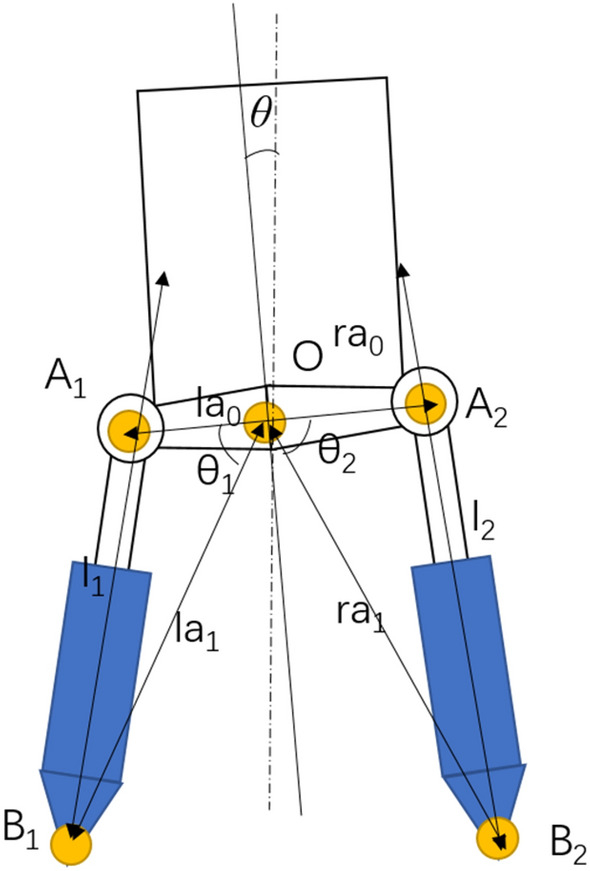
The swing schematic.

As is shown in Fig. 3, ignoring manufacturing tolerances, when $\theta { = 0}$, $\angle {\text{A}}_{{1}} {\text{OB}}_{{1}} { = }\angle {\text{A}}_{2} {\text{OB}}_{2} { = }\theta_{0}$. Suppose that $\angle {\text{A}}_{{1}} {\text{OB}}_{{1}} { = }\theta_{1}$, $\angle {\text{A}}_{2} {\text{OB}}_{2} { = }\theta_{2}$ ,during the rotating process, the length of the hydraulic cylinder can be obtained by the law of cosines

3 $$l_{1} = \sqrt {l_{a1}^{2} + l_{a0}^{2} - 2l_{a1} l_{a0} \cos \theta_{1} }$$

4 $$l_{2} = \sqrt {l_{a1}^{2} + l_{a0}^{2} - 2l_{a1} l_{a0} \cos \theta_{2} }$$

where $$\theta_{1} = \theta_{0} - \theta$$, $$\theta_{2} = \theta_{0} { + }\theta$$.

**Figure 4:**
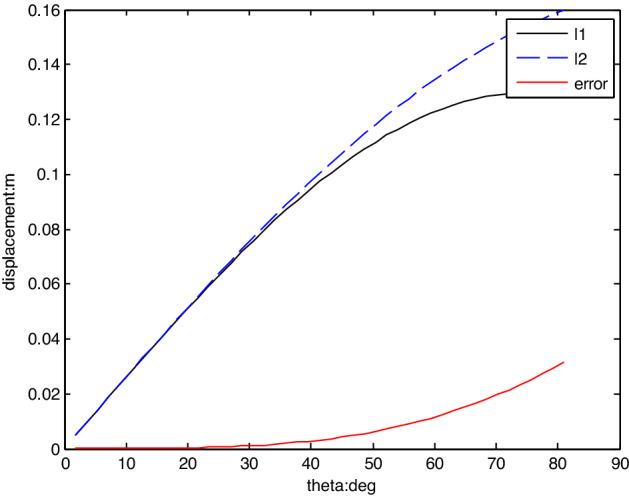
l_1_ and l_2_ when the swing rotates. (Kinematics model of the double actuated swing).

The result calculated using the MATLAB program is shown in Fig. 4. The lengths l_1_ and l_2_ are small and exhibit similar amplitudes when the swing DOF varies from the centre position θ = 0°to the θ = 60° position.

5 $${\text{A}}_{1} l_{1} \sin \theta_{1} {\text{ + A}}_{2} l_{2} \sin \theta_{2} \approx {\text{A}}_{2} l_{1} \sin \theta_{1} + {\text{A}}_{1} l_{2} \sin \theta_{2} \approx {\text{A}}_{{\text{s}}}$$

and

6 $$\Delta l_{1} \approx \Delta l_{2} \approx y,$$

Subsequently, (2) can be written as

7 $$(P_{1} - P_{2} )A_{s} = 2m\ddot{y} + I\ddot{\theta } + {\text{C}}\dot{\theta }$$

Considering leakage and compressibility, the dynamics of the cylinder oil flow can be expressed as^17^

8 $$\begin{gathered} \frac{{V_{1} \dot{P}_{1} }}{{\beta_{e} }} = - A_{s} \dot{x}_{l} + Q_{1} - C_{t} (P_{1} - P_{2} ) \hfill \\ \frac{{V_{2} \dot{P}_{2} }}{{\beta_{e} }} = A_{s} \dot{x}_{l} - Q_{2} + C_{t} (P_{1} - P_{2} ) \hfill \\ \end{gathered}$$

where C_t_ is the internal leakage coefficient, V_1_ and V_2_ denote the total fluid volumes of the hydraulic cylinders, *β*_e_ is the bulk modulus of the fluid, and Q_1_ and Q_2_ are the fluid flow rates of the cylinders. The displacement of the spool valve *x*_v_ is

9 $$\begin{aligned} & Q_{1} { = }k_{q} x_{v} \sqrt {\Delta P_{1} } \\ & Q_{2} { = }k_{q} x_{v} \sqrt {\Delta P_{2} } \\ \end{aligned}$$

where k_q_ is the flow gain coefficient of the servo valve.

The dynamics of servo valve have been incorporated in the controller design. Here, the valve dynamics are neglected and the servo valve opening *x*_*v*_ is proportional to the control input, since a high-response servo valve is used.

10 $$x_{v} = k_{a} u$$

where k_a_ is the servo amplifier gain and u is the servo valve control input signal.

The state variables are defined as

11 $${\mathbf{x}} = \left[ {\begin{array}{*{20}c} {x_{1} } & {x_{2} } & {x_{3} } & {x_{4} } \\ \end{array} } \right] = \left[ {\begin{array}{*{20}c} y & {\dot{y}} & {P_{1} } & {P_{2} } \\ \end{array} } \right]$$

According to (7–10), the dynamics are expressed in a state-space form as

12 $$\begin{aligned} & \dot{x}_{1} = x_{2} \\ & \dot{x}_{2} = \frac{1}{M}\left[ {(x_{3} - x_{4} )A_{s} - Bx_{2} - d} \right] \\ & \dot{x}_{3} = \frac{{\beta_{e} }}{{V_{0} }}\left( { - A_{s} x_{2} + k_{q} k_{a} \sqrt {\Delta P_{1} } u - C_{t} (x_{3} - x_{4} )} \right) \\ & \dot{x}_{4} = \frac{{\beta_{e} }}{{V_{0} }}\left( {A_{s} x_{2} { - }k_{q} k_{a} \sqrt {\Delta P_{2} } u{ + }C_{t} (x_{3} - x_{4} )} \right) \\ \end{aligned}$$

where B is the damping coefficient for cylinder, V_0_ is the total fluid volume of the two cylinders V_0_ = V_1_ = V_2_, and A_s_ is the total area of the cylinders.

M is the equivalent load mass obtained from the three terms at the right side of Eq. (7). M can be acquired by the $M\ddot{l}_{1} = I\ddot{\theta }$ and the second order differential of (3) or (4). As M does not vary greatly when $\left| \theta \right| < 60^{ \circ }$ , for simplify reason, M is set as a constant in the simulation experiment.

The time derivative of the second expression in (12) is

13 $$\dddot y = \frac{1}{M}\left[ {(\dot{x}_{3} - \dot{x}_{4} )A_{s} - B\dot{x}_{2} - \dot{d}} \right]$$

In (8), the difference in the third and fourth terms is

14 $$\begin{aligned} \dot{x}_{3} - \dot{x}_{4} = & \frac{{\beta_{e} }}{{V_{0} }}[( - 2A_{s} x_{2} + k_{q} k_{a} (\sqrt {\Delta P_{1} } + \sqrt {\Delta P_{2} } )u \\ & - 2C_{t} (x_{3} - x_{4} )] \\ \end{aligned}$$

Thus,

15 $$\begin{aligned} & \dddot y = - \frac{{2\beta_{e} A_{s}^{2} x_{2} }}{{MV_{0} }} - \frac{{2\beta_{e} A_{s} }}{{MV_{0} }}C_{t} (x_{3} - x_{4} ) - \frac{B}{M}\dot{x}_{2} - \frac{{\dot{d}}}{M} + \\ & \frac{{\beta_{e} }}{{MV_{0} }}k_{q} k_{a} (\sqrt {\Delta P_{1} } + \sqrt {\Delta P_{2} } )u \\ \end{aligned}$$

### Controller design

The following unknown parameter set is defined:

$$\sigma_{1} { = } - \frac{{2\beta_{e} A_{s}^{2} }}{{MV_{0} }},\sigma_{2} { = } - \frac{{2\beta_{e} A_{s} C_{t} }}{{MV_{0} }},\sigma_{3} = - \frac{B}{M},\sigma_{4} { = } - \frac{1}{M},\sigma_{5} = \frac{{\beta_{e} }}{{MV_{0} }}k_{q} k_{a}$$

The state-space Eq. (15) is linearly parameterized as^40^

16 $$\begin{aligned} & f = \sigma_{1} x_{2} + \sigma_{2} \left( {x_{3} - x_{4} } \right){ + }\sigma_{3} \dot{x}_{2} + \sigma_{4} \dot{d} \\ & g = \sigma_{5} (\sqrt {\Delta P_{1} } + \sqrt {\Delta P_{2} } ) \\ \end{aligned}$$

Function *f* can be expressed as $f = \hat{f} + \Delta f$, where $\hat{f}$ is the nominal part, and Δ*f* is the uncertain part, bounded as

17 $$\left| {\Delta f({\mathbf{x}},t)} \right| \le F({\mathbf{x}},t)$$

18 $$\hat{f} = \hat{\sigma }_{1} x_{2} + \hat{\sigma }_{2} \left( {x_{3} - x_{4} } \right){ + }\hat{\sigma }_{3} \dot{x}_{2} + \hat{\sigma }_{4} \dot{d}$$

where F is a known function.

The control gain g is confined to a certain constant range.

19 $$0 < \beta_{\min } < g < \beta_{\max }$$

The estimated value of g is

20 $$\hat{g} = \hat{\sigma }_{5} (\sqrt {\Delta P_{1} } + \sqrt {\Delta P_{2} } )$$

The control objective is to ensure that *y* asymptotically tracks the desired trajectory *y*_d_.

The tracking error is defined as

21 $$\tilde{y} = y - y_{d}$$

The sliding mode surface is defined as

22 $$s = \left( {\frac{d}{dt} + \lambda } \right)^{2} \tilde{y} = \ddot{\tilde{y}} + 2\lambda \dot{\tilde{y}} + \lambda^{2} \tilde{y}$$

Differentiating s with respect to t yields

23 $$\begin{aligned} \dot{s} = & \dddot y - \dddot y_{d} + 2\lambda \ddot{\tilde{y}} + \lambda^{2} \dot{\tilde{y}} \\ = & f + gu - \dddot y_{d} + 2\lambda \ddot{\tilde{y}} + \lambda^{2} \dot{\tilde{y}} \\ \end{aligned}$$

The Lyapunov function candidate is defined as

24 $$V(s) = \frac{1}{2}s^{2}$$

Thus,

25 $$\begin{aligned} \dot{V} = & s\dot{s} = s\{ \sigma_{1} x_{2} + \sigma_{2} (x_{3} - x_{4} ) + \sigma_{3} \dot{x}_{2} + \sigma_{4} \dot{d} \\ & + \left[ {\sigma_{5} (\sqrt {\Delta P_{1} } + \sqrt {\Delta P_{2} } )u - \dddot y_{d} + 2\lambda \ddot{\tilde{y}} + \lambda^{2} \dot{\tilde{y}}} \right]\} \\ \end{aligned}$$

The control input u is

26 $$\begin{aligned} & u = \frac{1}{{\hat{g}}}(\hat{u} - v) \\ & \hat{u} = - \hat{f} + \dddot y_{d} - 2\lambda \ddot{\tilde{y}} - \lambda^{2} \dot{\tilde{y}} \\ & v = ksat\left( {\frac{s}{\phi }} \right) \\ \end{aligned}$$

where f is the boundary layer, k is the control gain, and sat(Δ) is the saturation function, defined as

27 $${\text{sat}}(\Delta ) = \left\{ {\begin{array}{*{20}c} {1,{\kern 1pt} \quad \Delta > 1,} \\ {\Delta ,\quad \left| \Delta \right| < 1,} \\ { - 1,\quad {\kern 1pt} \Delta < - 1,} \\ \end{array} } \right.$$

To obtain the adaptation parameters, the following Lyapunov function is defined:

28 $$V_{1} (s) = \frac{1}{2}s^{2} + \frac{1}{2}\sum\limits_{i = 1}^{5} {\gamma_{i} \hat{\sigma }_{i}^{2} }$$

where $\hat{\sigma }_{i}$ is the estimated value of $\sigma_{i}$.

The estimation error is

29 $$\tilde{\sigma }_{i} { = }\sigma_{i} - \hat{\sigma }_{i}$$

The time derivative of V_1_ is

30 $$\begin{aligned} \dot{V}_{1} = & s\dot{s}{ + }\sum\limits_{i = 1}^{5} {\gamma_{i} \hat{\sigma }_{i} \dot{\hat{\sigma }}_{i} } = - \hat{\sigma }_{1} \left[ {s\sigma_{1} x_{2} { - }\gamma_{1} \dot{\hat{\sigma }}_{1} } \right] \\ & + \hat{\sigma }_{2} \left[ {s\left( {x_{3} - x_{4} } \right) - \gamma_{2} \dot{\hat{\sigma }}_{2} } \right] + \hat{\sigma }_{3} (\dot{x}_{2} - \gamma_{3} \dot{\hat{\sigma }}_{3} ) \\ & { + }\hat{\sigma }_{4} ({\text{s}}\dot{\hat{d}} + \gamma_{4} \dot{\hat{\sigma }}_{4} )\left[ {\sigma_{5} (\sqrt {\Delta P_{1} } + \sqrt {\Delta P_{2} } )u - \gamma_{5} \dot{\hat{\sigma }}_{5} } \right] \\ & - ksat\left( {\frac{s}{\phi }} \right)s + \sigma_{4} \dot{\hat{d}}s \\ \end{aligned}$$

To ensure that $\dot{V}_{1} < 0$ , the adaptation laws are set as

31 $$\begin{aligned} & \dot{\hat{\sigma }}_{1} = \Pr oj_{{\hat{\sigma }_{1} }} \left( { - \frac{{s\sigma_{1} x_{2} }}{{\gamma_{1} }}} \right) \\ & \dot{\hat{\sigma }}_{2} = \Pr oj_{{\hat{\sigma }_{2} }} \left( { - \frac{{s\left( {x_{3} - x_{4} } \right)}}{{\gamma_{2} }}} \right) \\ & \dot{\hat{\sigma }}_{3} = \Pr oj_{{\hat{\sigma }_{3} }} \left( {\frac{{\hat{\sigma }_{3} \dot{x}_{2} }}{{\gamma_{3} }}} \right) \\ & \dot{\hat{\sigma }}_{4} { = }\Pr oj_{{\hat{\sigma }_{4} }} \left( { - \frac{{{\text{s}}\dot{\hat{d}}}}{{\gamma_{4} }}} \right) \\ & \dot{\hat{\sigma }}_{5} = \Pr oj_{{\hat{\sigma }_{5} }} \left( {\frac{{\sigma_{5} (\sqrt {\Delta P_{1} } + \sqrt {\Delta P_{2} } )us}}{{\gamma_{5} }}} \right) \\ \end{aligned}$$

Function Proj() is a discontinuous projection, defined as

32 $$\Pr oj_{{\hat{\sigma }_{{\text{i}}} }} ( \bullet ) = \left\{ {\begin{array}{*{20}l} {0{\kern 1pt} } \hfill & {{\kern 1pt} if\quad \hat{\sigma }_{{\text{i}}} = \sigma_{{{\text{imax}}}} \& \bullet > 0,} \hfill \\ 0 \hfill & {if\quad {\kern 1pt} \hat{\sigma }_{{\text{i}}} = \sigma_{{{\text{imin}}}} \& \bullet < 0,} \hfill \\ \bullet \hfill & {otherwise} \hfill \\ \end{array} } \right.$$

For any adaptation function, the projection mapping ensures that

33 $$\hat{\sigma }_{{\text{i}}} \in \left\{ {\hat{\sigma }_{{\text{i}}} :\sigma_{{{\text{imin}}}} \le \hat{\sigma }_{{\text{i}}} \le \sigma_{{{\text{imax}}}} } \right\}$$

34 $$\hat{\sigma }_{{\text{i}}} \left[ {\gamma_{{\text{i}}} proj_{{\hat{\sigma }_{{\text{i}}} }} (\gamma_{{\text{i}}}^{ - 1} \Delta_{i} ) - \Delta_{i} } \right] \le 0$$

Controller (26) and adaptation laws (31) ensure that the tracking errors asymptotically converge to zero, i.e., $\tilde{x}(t) \to 0$ as time $t \to \infty$.

## Identification of the moment of inertia

### Volume and shape identification by stereo vision

To get the moment of inertia of the object, the volume and shape is needed. For the experiment in the paper, a simple method by corner and depth for a rectangle stone object is used.

**Figure 5:**
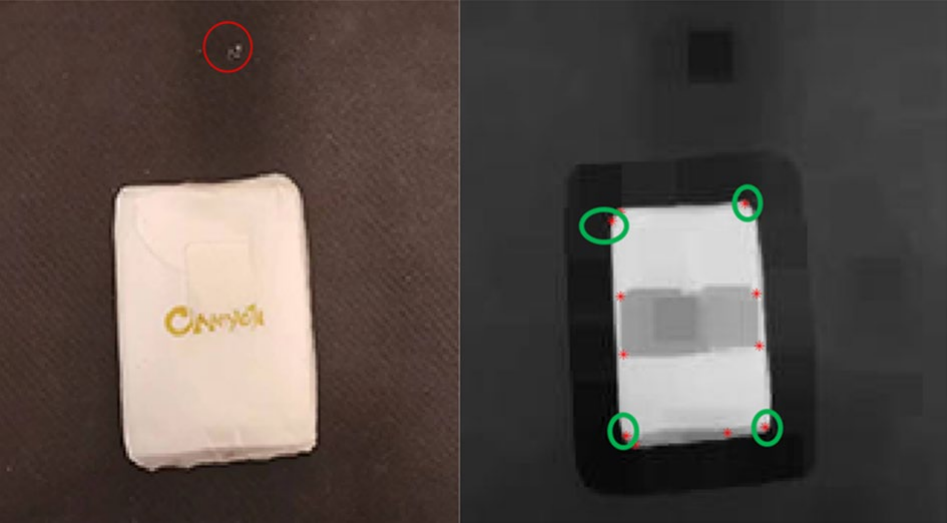
The corner extracting.

Figure 5 shows an image from a scenario that the stereo camera is placed at the top of the object, a stone covered by write papers as the work object is placed at the floor with black background.

Erode algorithm is applied to to remove the interference of small targets other than the targets. For example, small spots in the red circle of the left image may bring disturbance in the following process. Binarize for the eroded image is used for the 2-D grayscale image by thresholding, thus ROI (range of interest) is obtained.

Harris algorithm is applied to the corner extracting process. The result is shown as the red points in the right image. According to the four points in the green circles, the length, width and position of the object are obtained.

Zhang’s calibration algorithm is used for Stereo calibration. After rectifying the pair of stereo images from the two cameras, BlockMatching disparity algorithm is used the get the depth of ROI^41^. Then the height of the object is obtained. Then the volume and shape of the object can be obtained.

**Figure 6:**
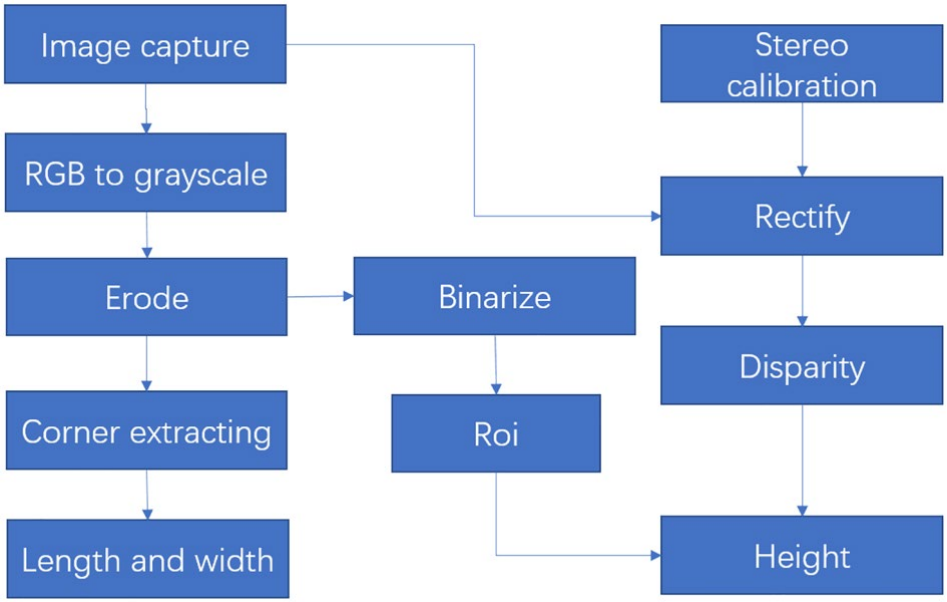
Image processing in computer vision.

Figure 6 shows the procedure of the image process. Similar methods can be used for cylindrical objects to obtain the diameter and length of the object through the corners.

### The moment of inertia to swing

The swing DOF when an object is being carried pertains to a robot arm system with constant parameters that cannot be identified offline. As described in "Sliding adaptive robust controller for the swing" section, the main parameters in (10) are dependent on the equivalent mass M. The equivalent mass M is composed of the mass of the swing hydraulic cylinder push rod and moment of inertia I. Therefore, I is

35 $$I = I_{a} + I_{b} + I_{o}$$

where I_a_ is the moment of inertia of the arm and folk glove, and I_b_ is the moment of inertia of the linkage system with the swing and boom. I_a_ and I_b_ can be accurately obtained through offline identification. I_o_ is the moment of inertia of the object being carried, which varies in a large range and cannot be accurately calculated offline. A new method is proposed that combines the stereo vision system and hydraulic cylinder information of the boom and arm to obtain I_o_.

The density ρ and approximate volume V of the working object can be derived as

36 $$I_{o} { = }\int {\rho {\text{V}}}$$

However, the shape and volume obtained by the vision system are considerably different from those of the work object, and thus, the direct use of these parameters may lead to a low estimation accuracy. The force obtained by the force sensor is highly accurate. If the mass of the work object can be obtained considering the force of the arm and boom, the moment of inertia can be corrected to obtain more accurate moment of inertia parameters.

**Figure 7:**
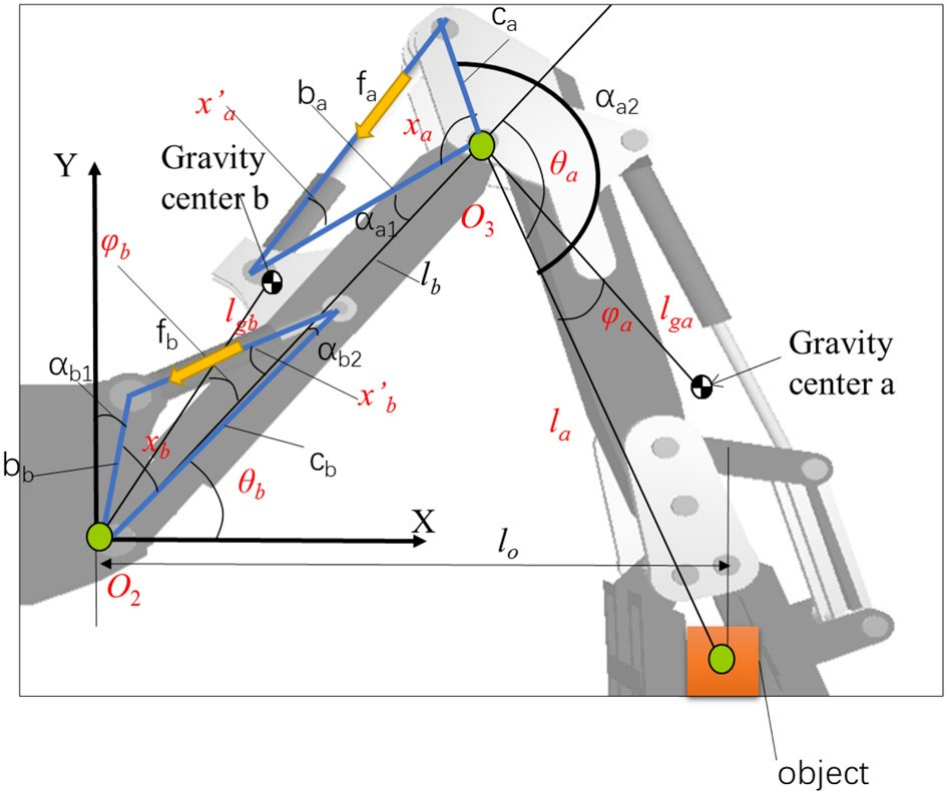
Boom and arm parameter.

The dynamic model for a system composed of booms, arms and folkgloves is^29^

37 $$\begin{aligned} \left[ {\begin{array}{*{20}l} {\tau_{b} } \hfill \\ {\tau_{a} } \hfill \\ \end{array} } \right] = & \left[ {\begin{array}{*{20}l} {m_{b} l_{gb} C_{b} + m_{a} l_{b} } \hfill & {m_{a} l_{ga} C_{a} } \hfill \\ 0 \hfill & {m_{a} l_{ga} C_{a} } \hfill \\ \end{array} } \right]\left[ {\begin{array}{*{20}l} {C\theta_{b} } \hfill \\ {C\theta_{ba} } \hfill \\ \end{array} } \right]{\mathbf{g}} \\ & -\left[ {\begin{array}{*{20}l} {m_{b} l_{gb} S_{b} } \hfill & {m_{a} l_{ga} S_{a} } \hfill \\ 0 \hfill & {m_{a} l_{ga} S_{a} } \hfill \\ \end{array} } \right]\left[ {\begin{array}{*{20}l} {S\theta_{b} } \hfill \\ {S\theta_{ba} } \hfill \\ \end{array} } \right]{\mathbf{g}} \\ \end{aligned}$$

where τ_a_ and τ_b_ denote the torques of the arm and boom, respectively, as shown in Fig. 7.

$$C_{b} = \cos \varphi_{b}$$, $$C_{{\text{a}}} = \cos \varphi_{a}$$, $$S_{b} = \sin \varphi_{b}$$, $$S_{a} = \sin \varphi_{a}$$, $$C\theta_{b} = \cos \theta_{b}$$, $$C\theta_{ba} = \cos (\theta_{b} - \theta_{a} )$$, $$S\theta_{b} = \sin \theta_{b}$$,$$S\theta_{ba} = \sin (\theta_{b} - \theta_{a} )$$.

The boom angle *θ*_*b*_ is

38 $$\theta_{b} = x_{b} - \alpha_{b1} - \alpha_{b2}$$

The arm angle *θ*_*a*_ is

39 $$\theta_{a} = x_{a} - \pi + \alpha_{a1} + \alpha_{a2}$$

The boom push force is

40 $$f_{b} = \frac{{\tau_{b} }}{{c_{b} \sin x^{\prime}_{b} }}$$

The arm push force is

41 $$f_{a} = \frac{{\tau_{a} }}{{c_{a} \sin \left( {x_{a} + x^{\prime}_{a} } \right)}}$$

When the robot carries an object, the torques can be formulated as,

42 $$\begin{aligned} \left[ {\begin{array}{*{20}c} {\tau_{b} } \\ {\tau_{a} } \\ \end{array} } \right] = & \left[ {\begin{array}{*{20}c} {\begin{array}{*{20}c} {m_{b} l_{gb} C_{b} + m_{a} l_{b} { + }m_{o} l_{o} } \\ 0 \\ \end{array} } & {\begin{array}{*{20}c} {(m_{a} l_{ga} + m_{o} l_{o} )C_{a} } \\ {(m_{a} l_{ga} + m_{o} l_{o} )C_{a} } \\ \end{array} } \\ \end{array} } \right]\left[ {\begin{array}{*{20}c} {C\theta_{b} } \\ {C\theta_{ba} } \\ \end{array} } \right]{\mathbf{g}} \\ & - \left[ {\begin{array}{*{20}c} {\begin{array}{*{20}c} {m_{b} l_{gb} S_{b} } \\ 0 \\ \end{array} } & {\begin{array}{*{20}c} {(m_{a} l_{ga} + m_{o} l_{o} )S_{a} } \\ {(m_{a} l_{ga} + m_{o} l_{o} )S_{a} } \\ \end{array} } \\ \end{array} } \right]\left[ {\begin{array}{*{20}c} {S\theta_{b} } \\ {S\theta_{ba} } \\ \end{array} } \right]{\mathbf{g }} \\ \end{aligned}$$

Thus, the following equations can be used to obtain m_o_

43 $$\begin{aligned} \tau_{a} { = } & (m_{a} l_{ga} + m_{o} l_{{{\text{g}}o}} )C_{a} C\theta_{ba} {\text{g}} \\ & - (m_{a} l_{ga} + m_{o} l_{go} )S_{a} S\theta_{ba} {\text{g}} \\ \end{aligned}$$

44 $$\begin{aligned} & \tau_{b} = \left( {m_{b} l_{gb} C_{b} + m_{a} l_{b} + m_{o} l_{o} } \right)C\theta_{b} {\mathbf{g}} \\ & \quad - m_{b} l_{gb} S_{b} S\theta_{b} {\mathbf{g}} - \left( {m_{a} l_{ga} + m_{o} l_{o} } \right)S_{ab} S\theta_{ba} {\mathbf{g}} \\ \end{aligned}$$

where L_go_ is the distance from the centre of the object to the swing, determined using the Digiclops system.

45 $$m_{o} = \frac{{\tau_{a} - m_{a} l_{ga} {\text{g}}\left( {C_{a} C\theta_{ba} - S_{a} S\theta_{ba} } \right)}}{{l_{go} {\text{g(}}C_{a} C\theta_{ba} - S_{a} S\theta_{ba} )}}{ = }m_{o1}$$

46 $$\begin{aligned} & m_{o} = \frac{{\tau_{b} - \left( {m_{b} l_{gb} C_{b} + m_{a} l_{b} } \right)C\theta_{b} {\mathbf{g}} + }}{{l_{o} {\mathbf{g}}\left( {C\theta_{b} - S_{ab} S\theta_{ba} } \right)}} \\ & \frac{{m_{b} l_{gb} S_{b} S\theta_{b} {\mathbf{g}} + m_{a} l_{ga} S_{ab} S\theta_{ba} {\mathbf{g}}}}{{}} = m_{o2} \\ \end{aligned}$$

Although m_o_ can be obtained using Eqs. (45) and (46), the two equations are slightly different. Equation (45) is relatively simple and must be used when possible. However, when the arm is vertical to the ground, singularity appears, and τ_a_ becomes extremely small. In this case, it is difficult to obtain the force. Therefore, the following formula is used to define m_o_

47 $$m_{o} = \left\{ {\begin{array}{*{20}l} {m_{o1} } \hfill & {\left| {\theta_{ab} } \right| < 20^{ \circ } } \hfill \\ {m_{o2} } \hfill & {else} \hfill \\ \end{array} } \right.$$

Using m_o_, a rough initial value of the moment of in can be obtained through the following equation:

48 $$I_{o1} = \rho \int_{{l_{o\min } }}^{{l_{o\max } }} {V_{1} dl}$$

where l_omin_ and l_omax_ denote the minimum and maximum distances of the object from the swing, respectively. The mass of the object is assumed to be evenly distributed, and volume V_1_ is determined through the vision information. I_o1_ can be used as the initial value of the parameter identification.

*Notice* The most remarkable feature of this paper is that the combination of gravity estimation algorithm and stereo vision algorithm can provide more accurate estimation of moment of inertia than the method based on robot dynamics alone.

### Discussion about the identification method combining with PD controller

The current robot joint control mostly adopts PD control because of its simple structure and excellent dynamic performance. Because of its phase advance ability, the dynamic response performance exceeds that of nonlinear control including sliding mode control in case of large deviation. But it also has many weaknesses, such as the large initial input, being sensitive to the friction force and to parameter uncertainty. PD nonlinear control algorithm which combines PD controller and nonlinear controller was proposed in many literatures^27,40^.

**Figure 8:**
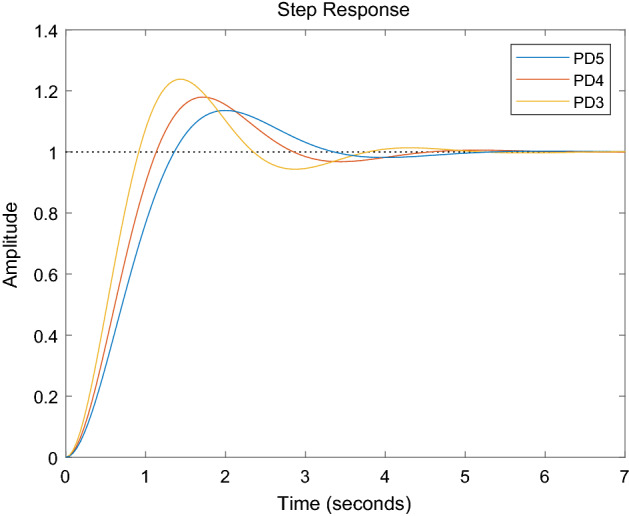
Effect of mass parameter on PD controller.

It is very easy to prove the effect of this method on PD controller. Figure 8 shows the closed loop step response of a common open-loop plant with transfer function

49 $${\text{G}} = \frac{1}{{0.5s^{2} + s}}$$

A PD controller is designed with the design goal of phase margin less than 15%. PD5 represents the closed-loop step response of a PD controller with the transfer function shown in (49).The close loop of the PD controller has a closed loop response of 55° phase margin and an overshoot of 13.6%.

Under the same target, with the accuracy of the mass parameter as 80% and 60% respectively, two PD controllers are designed with the following transfer functions.

50 $${\text{G}} = \frac{1}{{0.4s^{2} + s}}$$

and

51 $${\text{G}} = \frac{1}{{0.3s^{2} + s}}$$

These PD controllers are denoted PD4 and PD3, respectively.The phase margin of PD4 is 50.1°and the overshoot is 17.9%. The phase margin of PD3 is 44.5°and the overshoot is 23.8%. This is because the actual mass parameters are larger than those designed by the controller, so the overshoot and phase angle margin are both unfavourable.

During the process of grasping and placing, the change of its moment of inertia is often more than twice that of the robot arm. It is difficult to set the moment of inertia of the identification close to the true value. At this time, the identification algorithm combining vision and gravity has more important significance.

## Simulation experiment

### Sine trajectory tracking simulation

**Figure 9:**
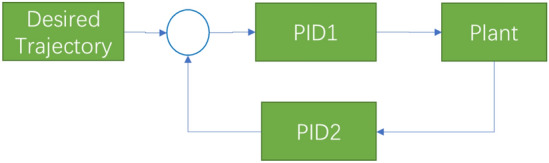
Compound PID controller (CPID).

**Figure 10:**
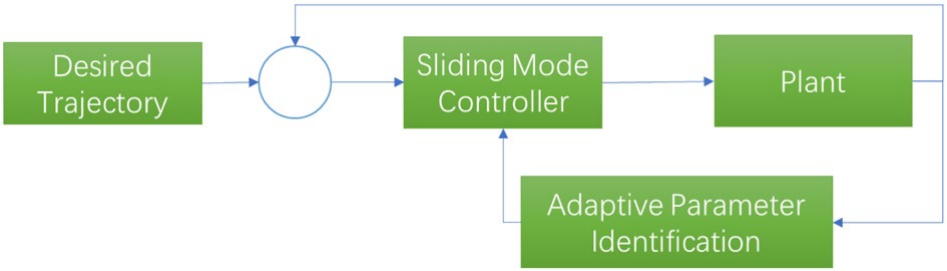
Sliding mode controller (SM).

**Figure 11:**
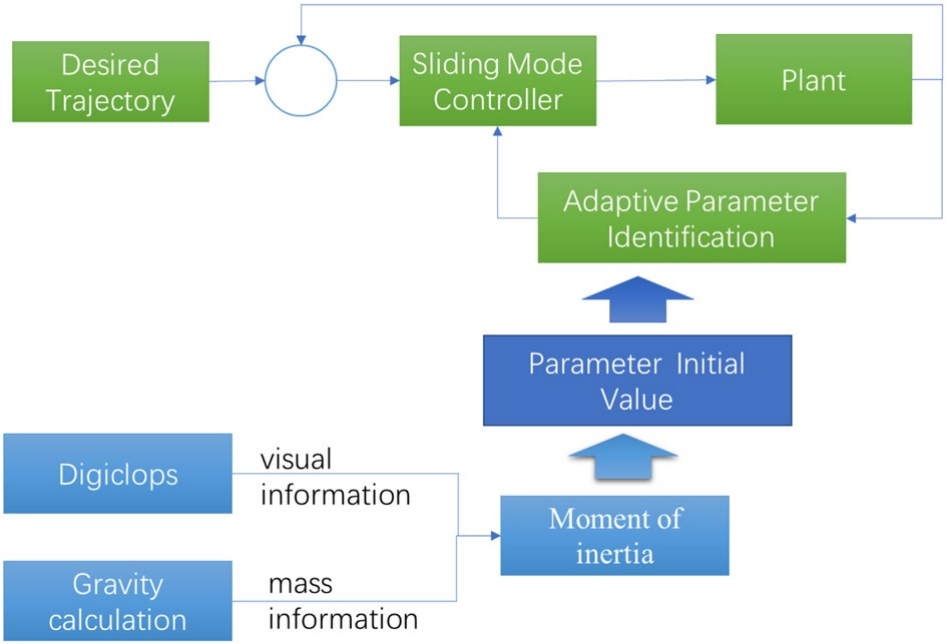
Sliding mode controller with initial value identification (SMI).

To verify the effect of the proposed scheme, the following simulation experiments are performed over the MATLAB Simulink platform. Three methods compound PID control (CPID), common sliding mode control (SM) and the proposed strategy, namely, sliding mode control with initial value (SMI), as shown in Figs. 9, 10 and 11, are used in the comparison experiments.

The CPID controller represents a practical controller in a linear system (Fig. 9). The zero and pole points are optimized through the feedforward and feedback PID controller, and the PID parameters can be adjusted in conjunction with the Routh stability criterion.

The sliding mode controller is shown in Fig. 10. The sliding mode controller with initial value identification is shown in Fig. 11.

The input modules on the desired trajectory output, such as the position, velocity and acceleration. The trajectory is y = sin(πt).

**Figure 12:**
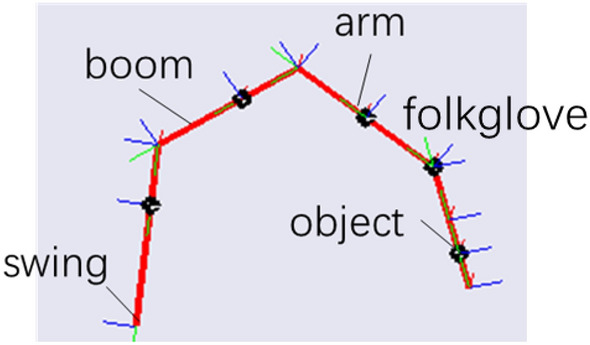
SimMechanics plant.

The plant built by SimMechanics in Fig. 12 is applied in the simulation program. The load features can be adjusted by setting the mass and gravity centre in the arm of the plant. Considering the work scenario, the load parameter is a constant value after it is determined. The commonly used friction force based on the LuGre model is introduced.

52 $$D = \upsilon_{0} z + \upsilon_{1} \frac{dz}{{dt}} + \upsilon_{2} \dot{\theta }$$

where z is the average deformation of the bristles, υ_0_ is the bristle stiffness coefficient, υ_1_ is the micro damping coefficient, and υ_2_ is the viscous coefficient. Random force disturbance ranges from ± 25N is added in the plant.

In order to obtain the guidelines for experimentation, the realistic model of the hydraulic parallel robot manipulator has been used. The system parameters are selected based upon their actual values and are given in Table 1.

**Table 1:** Parameters in the simulation program.

Parameter	Description	Value	Unit
A_s_	Area of the piston	6.08 × 10^3^	m^2^
β_e_	Bulk modulus of the fluid	690	MPa
w	Servo valve area gradient	0.008	m
Ps	Supply pressure	2	MPa
V_0_	Total fluid volume	9.05 × 10^–4^	m^3^
C_t_	Discharge coefficient of the cylinder	7 × 10^–9^	m^5^/(N s)
k_q_	Pressure gain	5.09 × 10^5^	m^4^s/kg
k_a_	Amplification gain	1.0	mA/V

In the simulation program, the real value of m_o_ is 2681 kg m^2^, and the calculated values of the parameters are.

53 $$\sigma = \left[ { - 4547, - 0.0113,0.2984,3.73 \times 10^{ - 4} ,14.48} \right]$$

The initial parameter value is input to the adaptive parameter identification module by setting the parameters in the S function. To validate the performance of the initial value identification, the initial values of M and σ are set. In SM, the initial value of m_o_ is set as the unloading moment of inertia, 723 kg m^2^; thus, the initial values of σ are

54 $$\sigma { = [} - {16861,} - {0}{\text{.0417,1}}{.1065,}0.0014{,53}{\text{.68]}}$$

In SMI, the initial value of m_o_ is set as 2469 kg m^2^; thus, the initial values of σ are

55 $$\sigma { = [} - {4937}{\text{.4,}} - {0}{\text{.0122,0}}{.3240,4}{\text{.05}} \times {10}^{{ - {4}}} {,15}{\text{.718]}}$$

The reference trajectory is θ_d_ = 60 × sinπt (°).

**Figure 13:**
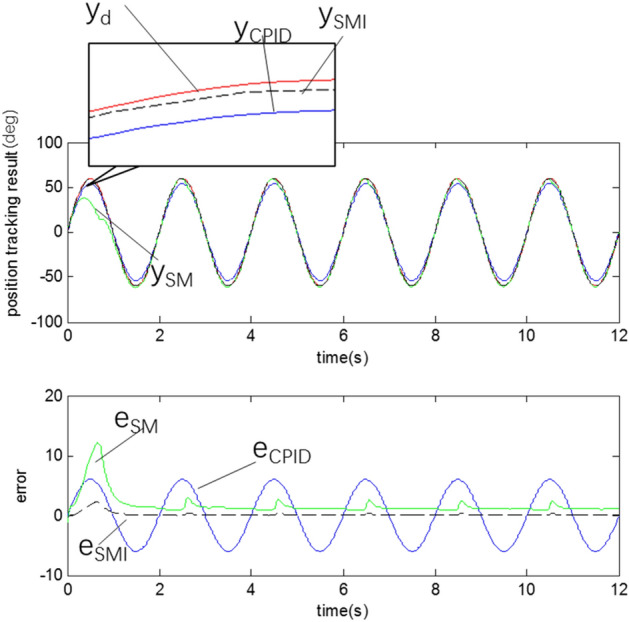
Trajectory tracking result.

According to the simulation experiment results shown in Fig. 13, the CPID method performs better at the beginning. But as the CPID does not have the parameter estimation function, the initial and final tracking errors are similar. Many shortcomings of PD controllers have been discussed in "Discussion about the identification method combining with PD controller" section, similar situation will happen to PID or CPID in this paper.

**Figure 14:**
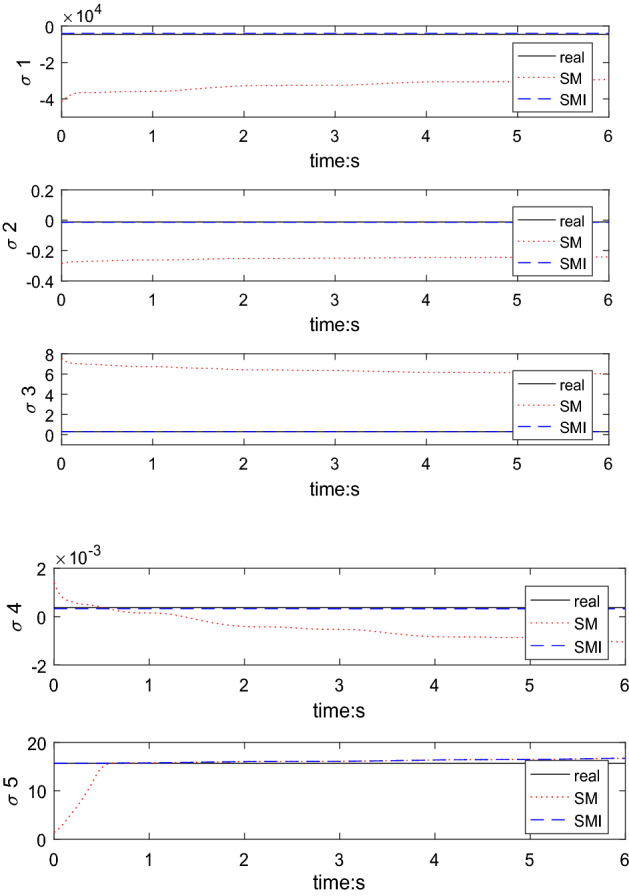
Parameter identification result.

The trajectory tracking error of the SM controller is considerably smaller than the steady-state error of the CPID and near to that of SMI. The controller can be considered to be very effective only in terms of trajectory tracking function. However, due to parameter coupling^14^, the parameters of the SM method cannot approach the target parameters, and thus, the performance of the controller is limited especially the initial performance. As is shown in Fig. 14.

The SMI trajectory tracking results in the later stage are the best among the three methods because of the superior parameter estimation, which was achieved with the proposed initial value estimation method. However, the parameter identification results are slightly different from the true values because of the accuracy limitations of the estimation algorithm.

### Carrying experiment simulation

The effect of the new parameter identification on PD controller has been described in "Discussion about the identification method combining with PD controller" section. Since PD controller and nonlinear controller can be designed and independently and the coupling of control can be realized by decoupling, to discuss the influence of initial value of SMI is enough. The step position response is used to simulate the object carrying process.

To validate the effect of initial moment of inertia on the proposed trajectory tracking control scheme during an object carrying task, a simulation in which a square wave represents the desired trajectory is performed.

Considering that the accuracy of the visual identification algorithm is affected by many factors, such as the grab position, the orientation of the target object, the uncertainty of the object's density and even the degree of illumination, to cover all the different situation is as impossible. This experiment only discussed the effect of identification accuracy, the following controller with different parameters are discussed.

1. SMI with the calculate value is described in "Moment of inertia identification experiment" section which represents the real value of the inertia parameter as SMI10.
2. A value of which the parameter is well identified as 0.8 times the mass, representing a smaller inertia parameter as SMI8.
3. For the identification result under bad conditions, the moment of inertia is 0.6 times of the calculated value as SMI6.

With the upper controllers, the carrying process with square wave as desired trajectory are simulated. the friction force and disturbance are the same as "Sine Trajectory tracking simulation" section.

**Figure 15:**
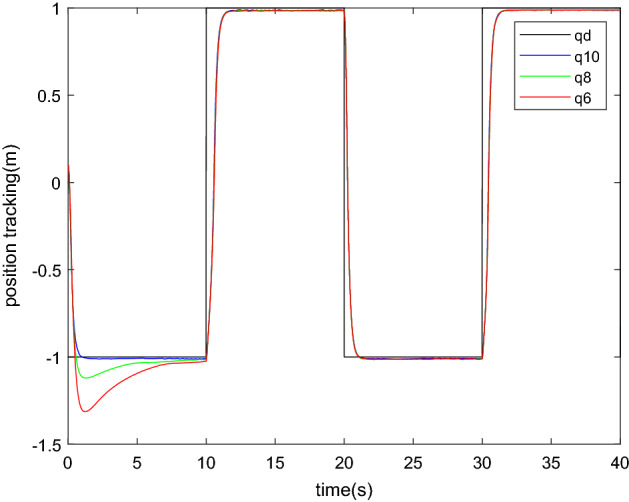
Position tracking result.

**Figure 16:**
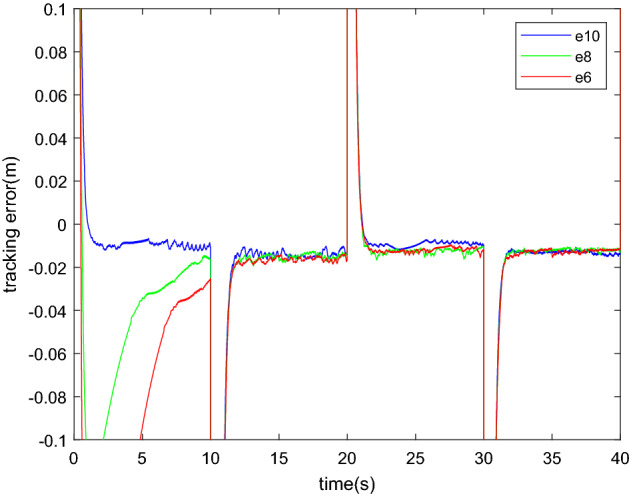
Tracking error.

From the result of Figs. 15 and 16, we can conclude that: The trajectory tracking performances of each controller are different at the first ten second. The accuracy mainly depends on the distance between the initial value and the true value. But after a certain period of time, each controller performs at almost the same accuracy. The influence of initial value is very small, especially after the step process. However, there is an error in the desired value due to imperfections in the identification algorithm that cannot be completely eliminated. The error is small, approximately 1% of the desired trajectory. Considering that the nonlinear algorithm mainly aims at the steady state situation, parameter changes and uncertainties, the effectiveness of the identification method in a sliding mode algorithm is proved.

During the processes of picking up and unloading objects of the carrying task, the mass parameters often change dramatically. At least two processes, carry and unload, are included and repeated. The initial value identification method can effectively improve the accuracy of the initial stage. The practical significance of the initial value identification method in this paper is proved. SMI is a practical method for the situation where the object changes frequently, but it has little impact on the situation that one constant load mass repeats many times.

The experimental results demonstrate the importance of the initial value identification method for all control strategies parameter identification. In addition to trajectory tracking, this method can be used in other fields, such as energy-saving control.

## Experiment

This section describes the moment of inertia identification experiment. The system setup and implementation issues are in "Experiment setup and implementation issues" section. The process and result of the identification experiment are in "Moment of inertia identification experiment" section.

### Experiment setup and implementation issues

The experimental setup consists of the following hardware components.

1. Construction robot reconstruction for the 10 T LiuGong excavator.
2. Pointgrey Research Colour Digiclops, with three Sony 1/3" progressive scan CCD cameras.
3. Rexroth Huade 4 WRE16-10 servo valve.
4. Druck PTX1400 pressure transmitters with an operating pressure range of 25 MPa.
5. Miran LVDT20 rod-type displacement sensor.
6. ADVANTECH PCI-1710UL Data acquisition card (DAQ) with 12-bit data acquisition and 100 kHz sampling rate.

The hardware of the control system consists of two PC-compatible computers that communicate through a local area network. All analogue measurement signals (cylinder position, chamber pressure, supply pressure and load force) are fed back to the slave PC through four plug-in DAQ cards.

### Moment of inertia identification experiment

To test the effect of the vision information combined with the gravity recognition algorithm of moment of inertia, as described in "Controller design" section, a three-dimensional vision and gravity compensation method was applied to grasp stone.

In the experiments with a 1.0 m long stone object, the object was grabbed in the middle position (0.5 m, denoted M) or edge position (0.9 m, denoted E), and the results are compared with the torque obtained using the robot dynamics method only and the vision method only.

The moment of inertia result is I_sum_. According to Table 2. The identification experiment results are shown in Table 2.

**Table 2:** Results of identification experiments.

	Calculated value	Identified value	Dynamic only	Vision only
M	2681	2668	2670	2348
E	2918	2867	2670	2557

In Table 2, the calculated value column shows the manually measured results, which has the highest accuracy. The identified value column shows the result with the method in this paper combined with stereo vision in "The moment of inertia to swing" section and robot dynamics in "The moment of inertia to swing" section. The dynamics only column shows the results calculated with only the robot dynamics method, and the vision only column shows the results calculated with only the stereo vision information. The volume is calculated according to the parameters from computer vision.

**Figure 17:**
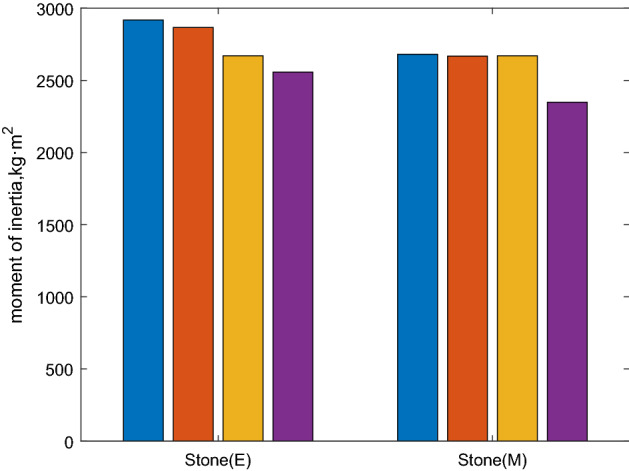
Results of identification experiments.

From Table 2 and Fig. 17, we can conclude that:

In this experiment, the moment of inertia of the load is three times that of the mechanical arm. The significance of identification algorithm of moment of inertia is validated.

Identified value with the method combined with stereo vision robot dynamic performs best of the three algorithms. This shows the superiority of the identification method of moment of inertia combined with vision that proposed in this paper.

Regarding the target object as a mass point, a moment of inertia can also be identified with the robot dynamic algorithm as Dynamic only. Dynamic only performs better than Vision only because the accuracy of force sensor is better than vision. But in the experiment, when the folkglove grasps the middle position and edge position of the stone, the results are the same. The robot dynamics algorithm cannot show the change of the grasp position.

Although the precision is low, Vision only is a very effective method. Considering the high price and complex structure of the force sensor and vision will be widely used in intelligent control, the method is still an effective method in the future.

Due to the influence of many factors on the visual algorithm, the relationship between its accuracy and the object needs to be further studied.

## Conclusion

This study focuses on a novel moment of inertia identification algorithm combined with stereo vision and robot dynamic and the adaptive robust sliding mode control scheme with the identified initial value for trajectory tracking of swing in a construction robot.

A novel control scheme that obtains the initial value of the moment of inertia of swing through the robot gravity identification algorithm and stereo vision information is proposed. The scheme can be used to overcome the limitations associated with the low convergence speed of the parameter identification algorithm and large change in the inertia caused by the change in the work object.

Simulation and online experiments are performed to validate the effect of the novel scheme. The sine trajectory tracking simulation experiment demonstrates the superiority of the SM algorithm over the CPID algorithm and necessity of obtaining the initial value for the sliding mode algorithm in SMI. Carrying simulation experiments involving the different accuracy validate the effectiveness of the identification method in a sliding mode algorithm. Moment of inertia identification experiment validated the feasibility and necessity of the new identification method. Many issues about the application of the identification method are discussed.

The method of identifying the moment of inertia based on the combination of stereo vision and robot dynamics proposed in this paper is applicable to all control strategies including mass parameter identification.

## Supplementary Information

Supplementary Information.

## Data Availability

The datasets supporting the conclusions of this article are included within the article.
