# Supplementary material for: Parameter adaptive sliding mode trajectory tracking strategy with initial value identification for the swing in a hydraulic construction robot
Source: Sci Rep. 2023 Apr 11;13:5855. doi: 10.1038/s41598-023-30952-x (PMC10090183; doi:10.1038/s41598-023-30952-x)
Supplement: Supplementary file 1 — Supplementary Information. [file 41598_2023_30952_MOESM1_ESM.docx]

Appendix

**Kinematic of the two cylinders in the swing**

As shown in Figure 1, ignoring manufacturing tolerances, when $\theta\text{=0}$,$\angle\text{A}_{1}\text{OB}_{1}\text{=}\angle\text{A}_{2}\text{OB}_{2}\text{=}\theta_{0}$. Assume that $\angle\text{A}_{1}\text{OB}_{1}\text{=}\theta_{1}$and $\angle\text{A}_{2}\text{OB}_{2}\text{=}\theta_{2}$when an object is being carried

$\theta_{1}\text{=}\theta_{0}\text{-}\theta$ (1)

$\theta_{2}\text{=}\theta_{0}\text{+}\theta$ (2)

The length of the hydraulic cylinder can be determined by the law of cosines

$l_{1}=\sqrt{l_{a1}^{2}+l_{a0}^{2}\text{-}2l_{a1}l_{a0}\cos\theta_{1}}$ (3)

$l_{2}=\sqrt{l_{a1}^{2}+l_{a0}^{2}\text{-}2l_{a1}l_{a0}\cos\theta_{2}}$ (4)


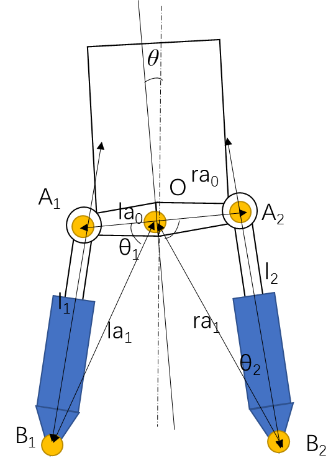


Figure S1 Schematic of the swing
